# Structural, electrical, and physical–mechanical properties of composites obtained based on filled polyolefins and thermoplastic elastomers

**DOI:** 10.1039/d5ra00105f

**Published:** 2025-02-27

**Authors:** Khayala Vagif gizi Allahverdiyeva, Najaf Tofig oglu Kakhramanov, Rena Vagif gizi Gurbanova

**Affiliations:** a Laboratory of Mechanical-Chemical Modification and Processing of Polymers, Institute of Polymer Materials of Ministry of Science and Education Republic of Azerbaijan Sumgait City Azerbaijan xayalaka4@gmail.com; b Chemistry and Inorganic Substances Technology Department, Azerbaijan State Oil and Industry University Baku City Azerbaijan

## Abstract

The paper presents the results of a study of the influence of various mineral and metal fillers, as well as carbon black and graphite on the structural features and quality of composite and nanocomposite materials based on polyolefins and their modifications. High density polyethylene (HDPE), low density polyethylene (LDPE), various copolymers of ethylene with α-olefins, polypropylene (PP), polypropylene random copolymer, block copolymer of ethylene with propylene, *etc.* were used as polyolefins. The filler used was mainly carbon black (CB) and graphite, as well as various natural minerals. The objective of the review material under consideration was to demonstrate promising possibilities for obtaining electrically conductive composite and nanocomposite materials based on dielectric polymers, which are included in the polyolefins. The introduction section examines the state of the problem of development and research of electrically conductive nanocomposites, as well as the goals and objectives of the research. The theoretical aspects of obtaining and studying composite materials based on polyolefins are considered, where the main attention is paid to studying the influence of filler content on the structural features and properties of polymer composites. Particular attention is paid to the compatibility of the mixture components and the establishment of the relationship between the filler particles and the macrochains of the polymer matrix. The significant role of compatibilizers in improving the compatibility of mixture components is shown. The article presents scientific provisions and theoretical background that explain the mechanism of the compatibilization process and its influence on the pattern of changes in electrical conductivity, physical, mechanical, thermophysical and thermal deformation properties of nanocomposites. Much attention is paid to the development of thermoplastic elastomers, taking into account the specifics of the interaction of thermoplastic (polyolefin) macrochains with various synthetic elastomers based on natural, butadiene-nitrile, butadiene–styrene and ethylene–propylene–diene rubbers. The influence of the thermoplastic-elastomer ratio, carbon black and graphite, and their modifications on the structure and properties of nanocomposites based on thermoplastic elastomers is considered. Significant attention is paid to the study of electrical conductivity of nanocomposites based on thermoplastic elastomers. It has been established that the degree of crystallinity of polyolefins and thermoplastic elastomers has a significant effect on the formation of the interspherulitic amorphous region in filled composites, which, in turn, has a primary effect on the mechanism of formation of tunnel and electron conductivity.

## Abstract 2

The paper presents the results of a study of the influence of various fillers on the structural features and quality of composite and nanocomposite materials based on polyolefins and their modifications.

## Graphical abstract

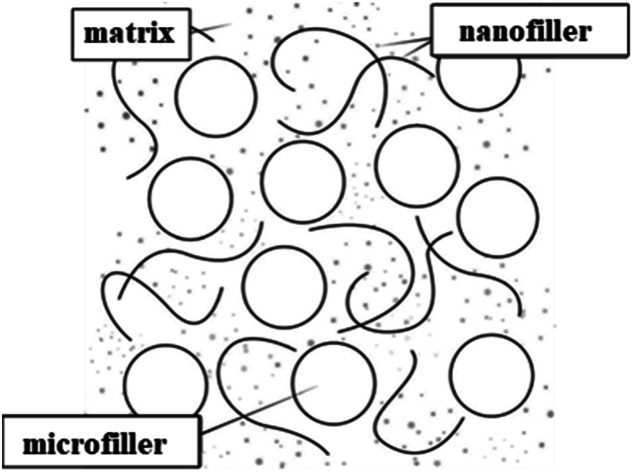

## 1. Introduction

With the development of industrial production techniques and technologies, interest in the development of qualitatively new types of polymeric materials capable of operating under harsh operating conditions has increased significantly. The use of various types of fillers, plasticizers, stabilizers and modifying additives has made it possible to satisfy, to varying degrees, the needs of customers in the sale of polymer composite materials (PCM).^1^ There are specific areas of application for polymeric materials that require the composite to have a number of useful properties at the same time. In this regard, the leader in the use of multifunctional polymers is the electronics industry.^2,3^ At this stage, the multifunctionality of PCM is one of the promising scientific directions, allowing to simultaneously solve several operational requirements when using it in specific areas of industry. We are talking about obtaining materials that have elasticity, high electrical conductivity, thermal conductivity, adhesion to metals, a set of necessary strength indicators, as well as satisfactory rheological parameters for processing by injection molding and extrusion.

In this regard, it should be noted that comparatively greater interest is generated by research aimed at obtaining and studying thermoplastic elastomers (TPEs), which have the properties of rubber, but are processed like thermoplastics. This is primarily due to the fact that rubber technology requires a lot of labor and time for its production, is characterized by low equipment productivity, and increased environmental impact in the workplace. In addition, rubber waste is practically not utilized in the recycling process.^4^ As a result of mixing plastics with elastomers, it is possible to obtain various types of TPE with high elastic-deformation properties. The resulting compositions have a highly elastic deformation region and are suitable for processing on high-performance equipment designed for thermoplastics. The use of multi-cavity molds and high-speed cycles of casting equipment not only improves the quality of materials, but also significantly reduces their cost.^5^ The use of nanoparticles as fillers has opened up new and reliable possibilities for the development and production of polymer nanocomposites with a unique combination of properties. The authors believe that in order to obtain and study electrically conductive nanocomposites based on dielectrics – polyolefins, a systematic approach to studying this problem should be taken. This approach includes an assessment of the role of the structure and degree of crystallinity of the polymer matrix, the type and content of nanoparticles, the compatibilizers used and other accompanying ingredients in changing the complex of physical and mechanical properties and the mechanism of formation of the crystalline structure and electrical conductivity of nanocomposites. It should be noted that when developing an electrically conductive material, the multifunctionality of the nanocomposite is of great importance. The multifunctionality of the nanocomposite includes, in addition to high electrical conductivity, good adhesive strength for attachment to a metal surface, high thermal conductivity to prevent overheating of the material during long-term use, wear resistance for use in contact with a rubbing surface, high strength, elasticity and the ability to be processed on high-performance equipment.^6^

In connection with the above, the purpose of this work was to use modern scientific approaches and theoretical provisions on the structure of polymeric materials, as well as methodological solutions for the development and study of composites, to show new possibilities for obtaining materials with predetermined properties.

## 2. Theoretical aspects of obtaining and studying composite materials based on polyolefins

The authors of works^6–8^ have been conducting scientific research for a long time on the study, processing, and investigation of composites and nanocomposites, dynamically vulcanized thermoplastic elastomers based on polyolefins (PO), intended for practical use in special areas of technology. In this case, a whole range of natural minerals such as vesuvianite, clinoptilolite, bentonite, graphite, carbon black, zeolite, quartz, calcite, aluminum hydroxide, talc, fibrous basalt, molybdenum sulfide, *etc.* were used as filler. Based on these minerals used in the composition of PO, a number of composite and nanocomposite polymeric materials have been obtained, characterized by high strength properties, electrical conductivity, heat resistance, friction, non-flammability, *etc.* For example, using vesuvianite, clinoptilolite, talc and quartz as fillers made it possible to obtain nanocomposites with high strength properties. It has been shown that the inclusion of molybdenum sulfide and graphite in the composition of the block copolymer of ethylene and propylene leads to the production of nanocomposites with high wear resistance and heat resistance.^9,10^ The use of aluminum hydroxide in the composition of various types of polyethylene mixtures has shown the possibility of obtaining fire-resistant materials on their basis for use in construction.^11–13^

The production of a relatively new generation of dynamically vulcanized polymer mixtures based on thermoplastic elastomers has shown that they have the properties of rubber, but at the same time can be processed like thermoplastics.^14^ The development of materials based on dynamically vulcanized nanocomposites that possess the properties of TPE with high strength, impact resistance and elasticity opens up new promising opportunities for their practical use in special areas of mechanical engineering.^15–18^

It should be noted that an important place is given to the study of technological features of processing nanocomposites based on PO by injection molding and extrusion methods. Conducting technological research allows us to optimize the temperature regime for processing nanocomposites and, thus, determine in advance the possibilities of obtaining materials with high physical, mechanical and operational properties based on them.^19^ Polyolefins are among the most multi-tonnage and in-demand among all types of polymers produced in industry, as they have high physical and mechanical properties and the ability to be processed using various methods. In addition, a distinctive feature of PO is their ability to be modified by various physical, physical–mechanical and chemical methods. From a technological point of view, the simplest and most effective way to modify PO is to introduce finely dispersed fillers into their composition.^20–26^ Therefore, the search for scientific and technical solutions aimed at simplifying PO processing and obtaining new types of composite materials (CM) with improved physical and mechanical properties is a pressing task. The introduction of various types of dispersed and fibrous fillers into the polymer matrix, along with a reduction in the cost of products obtained on their basis, makes it possible to obtain composites with predetermined physical, mechanical and thermal physical properties.^27–31^

**Fig. 1:**
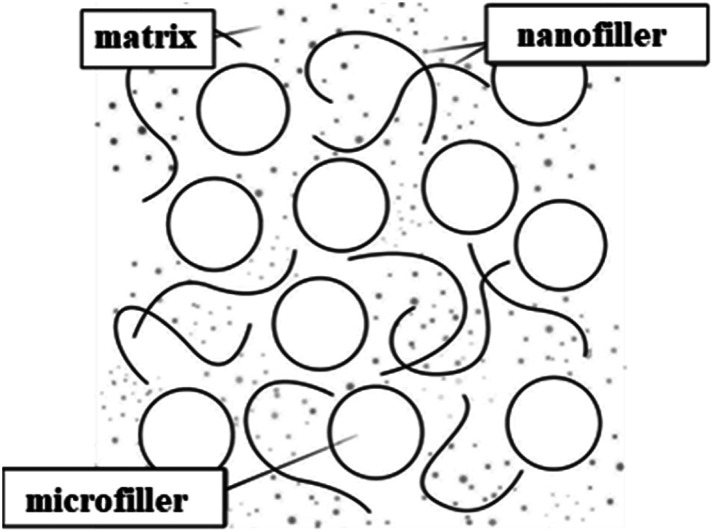
Morphology of micro- and nanofilled composites.^32^

**Fig. 2:**
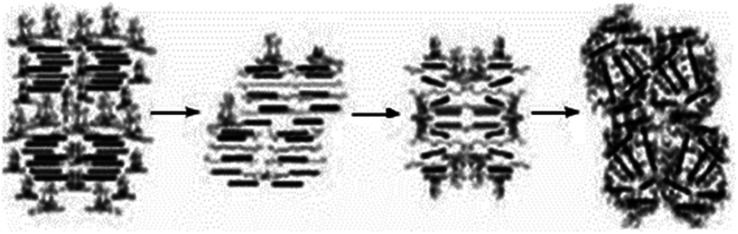
Scheme of polymer nanocomposite formation.^38^

In recent years, more extensive and intensive research has been conducted on modifying the structure and properties of polymers by introducing dispersed nanosized fillers into their composition. Fig. 1 shows a schematic representation of the morphology of a polymer composite (PC) filled with micro- and nanofillers.^32^ Originally, the term “PC” referred to materials consisting of various types of short and continuous fibers bonded to an organic polymer matrix. They were processed and manufactured to efficiently transfer loads between phases and to provide strength and high rigidity at relatively low levels. Thus, their initial goal was to obtain PC with high physical and mechanical properties. Nowadays, the term “PC” has been refined and, despite some inaccuracies, is generally used to describe materials consisting of one or more fillers or polymer blends.^33,34^ A nanocomposite is a multicomponent material consisting of several different phases. In this case, at least one of the phases must have a nanometer size.^35–37^ The formation of a polymer nanocomposite can be schematically depicted in the following figure (Fig. 2).^38^ In addition to the development of physical and mechanical properties, other specific properties can be achieved during the processing of polymer nanocomposites.

In addition to improving the physical and mechanical properties of polymer nanocomposites, other specific properties can be improved directly during the processing process. For example, thermal or electrical conductivity, low flammability, chemical resistance, *etc.*^39–42^ The synergistic effect that can arise when mixing fillers with a polymer matrix can lead to an additional increase in the physical and mechanical characteristics of the polymer nanocomposite.^43–45^ The use of mineral fillers as nanoparticles has been widely demonstrated in scientific studies cited in ref. 46 and 47. The use of heat-resistant mineral and metal nanoparticle fillers in the polymer matrix has opened up the prospect of using nanocomposites in harsh extreme operating conditions. High-speed injection of the melt into the mold during the injection molding process contributed to the formation of nanocomposites with high strength characteristics and less susceptibility of the polymer matrix to mechanical and thermal destruction.^48,49^

In general, the properties of filled polymer composites are formed in the process of complex influence of several factors. The most important of these are: the nature of the thermoplastic, the type of filler, their shape and size, as well as the change in local density depending on the volume of the sample.^50–54^ The influence of the type of thermoplastic and filler used on the process of forming the structure and properties of the composite is primarily determined by their technological compatibility and miscibility. If the thermoplastic and filler are incompatible, the resulting composite usually has low physical and mechanical properties. Thus, due to the effect of the applied load, the adhesive bonds between the polymer and the specific surface area of the filler particles are broken.^55–57^ The smaller the size of the filler particles, the larger its specific surface area and, accordingly, the higher the efficiency of adhesive contact with the polymer matrix. If the adhesion in the polymer–filler system in a composite is high, then the applied load will be uniformly distributed in the interphase region, which will ultimately contribute to obtaining composites with relatively high physical and mechanical properties.^58–65^

From the above it follows that the amount of filler is a determining factor in the creation of PC. As a rule, with an increase in the amount of fillers, the thermal stability, heat resistance, fire resistance, tensile strength, hardness and impact toughness of the composite increase. If the filler is dispersed, then with an increase in its quantity, the anisotropy of the physical and mechanical properties of the composite decreases.^66^ At the same time, the use of conventional fillers leads to an increase in the brittleness temperature, a decrease in strength properties and an increase in the viscosity of the composite melt. For this reason, the search for fillers capable of eliminating the above-mentioned shortcomings and improving the physical, mechanical and technological properties of the composite has remained a pressing scientific and technical problem over the past decades.

Hybrid materials with excellent structural and functional properties can be obtained by introducing nanofillers into polymer matrices. Polyhedral oligomeric silsesquioxane (POSS) nanoparticles have recently attracted much attention due to their nanometric size, the ease with which these particles can be incorporated into polymeric materials, and the unique ability to strengthen polymers. In ref. 67, the state of polymer nanocomposites containing POSS is considered. The influence of functional groups and POSS concentration on the polymer matrix during chemical crosslinking or physical mixing on the structure of nanocomposites is discussed. Also, in the work ref. 68 it is shown that the introduction of POSS into polymer matrices improves their thermal, mechanical, thermomechanical, dielectric and morphological properties, and also increases the resistance to moisture, corrosion, oxidation, ultra violet radiation and microbial organisms of the obtained polymer nanocomposites.

## 3. Development and research of composite materials based on thermoplastic elastomers

**Fig. 3:**
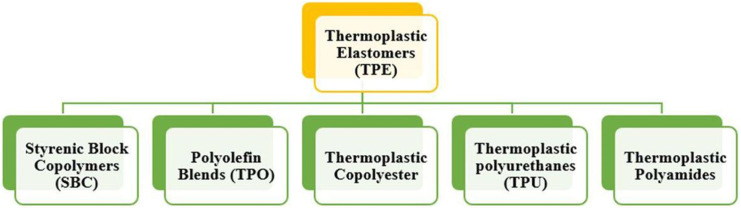
Types of thermoplastics elastomers.^70^

Thermoplastic elastomers are a rapidly developing area of polymer science and industry. Their distinctive feature is the ability to be processed on equipment designed for thermoplastics, while maintaining the characteristic physical and mechanical properties of rubber. When it is mentioned that TPE has the properties of rubber, what is meant is their ability to undergo highly elastic deformation, which can be established by studying thermomechanical properties. These properties of TPE provide two advantages over conventional rubbers: processing is faster and the resulting production waste, as in the case of thermoplastics, can be recycled.^69^ There are several types of TPE, which are illustrated in Fig. 3.^70^ TPEs based on PO and elastomers are the most promising materials in terms of production and practical use. This is due to the fact that they have good resistance to bending and friction, a wide range of operating temperatures, resistance to alkalis, mineral acids, water and polar organic solvents, and also have good electrical properties.^71,72^ It should also be noted that, compared to rubber, TPEs allow the use of modern processing methods characterized by high productivity, especially in the production of structural products with complex configurations.^73^

In ref. 74, PCs based on TPE were developed and manufactured. Isotactic PP and triple ethylene–propylene–diene elastomer (EPDM) were used as starting materials for mechanochemical synthesis. The diene component in EPDM is ethylidene norbornene, used at 4–5%. Dynamic vulcanization of the elastomer was carried out in the presence of sulfur. Dimethylpolysiloxane silica gel with a content in the range of 10–80 wt% was used as a filler. The maximum possible amount of filler in the matrix was 80 wt%. The introduction of a larger amount of filler into the polymer matrix creates difficulties when mixing them. As a result of introducing filler from 10 wt% to 70 wt%, such properties of composites as tensile strength, flexural strength and elastic modulus increase. However, when the amount of filler in the composite is 80 wt%, these indicators deteriorate significantly. Thus, it has been shown that the maximum filler content for the EPDM elastomer is 70 wt%.

The influence of glass fiber reinforced polyester composite on its physical, mechanical and thermal properties was investigated.^75^ A mixture of styrene–ethylene–butylene–styrene (SEBS) and maleated styrene–ethylene–butylene–styrene copolymer (SEBS-MA) was used as TPE. The components were mixed in a melt process and processed using injection molding. In this case, SEBS-MA was used as a compatibilizer. A study of the physical and mechanical properties of glass fiber-reinforced composites showed that the inclusion of SEBS-MA made it possible to improve the plasticity of TPE and increase the Izod impact strength. Scanning electron microscope (SEM) analysis of the structure showed that SEBS-MA together with glass fiber is dispersed in a polyester matrix. The results of differential scanning calorimetry (DSC) showed that the inclusion of SEBS-MA in the matrix led to a decrease in its crystallinity.

To obtain materials with higher density and elasticity than isotactic polypropylene (PP), composites were developed based on its mixture with ethylene–octene copolymer in the presence of fillers such as talc, mica, barium sulfate (BaSO_4_).^76^ Depending on the composition of the composite, the melt flow index (MFI), density, bending and stretching, impact resistance, hardness, moisture and water absorption properties of the mixture were studied. It was determined that the density of PP increased by 45% due to the addition of 12 vol% BaSO_4_. In addition to fillers, the use of TPE as a polymer matrix leads to the elimination of rigidity and increased impact resistance. This makes it possible to maintain the elasticity of the composite even when filler is introduced. In this case, it was found that the composite containing BaSO_4_ had a flexural modulus corresponding to that of PP/TPE and impact properties superior to PP and PP/TPE blends. Good results on moisture and water absorption of composites were obtained for samples containing BaSO_4_.

Mixing SEBS-MA and functionalized graphene (FG) in tetrahydrofuran resulted in SEBS-MA/FG nanocomposites with specific properties.^77^ The resulting composites were characterized by high strength and physical and mechanical properties. Thus, SEBS/FG composites are promising materials as TPEs and can be recommended for use as sealants in the automotive industry.^78^ In this work, SEBS and PP were mixed and ground at low temperatures. It was found that SEBS, which contains styrene segments and has a high molecular weight, does not melt and flows poorly under the influence of laser beams, *i.e.* is not suitable for 3D printing. Low molecular weight SEBS is suitable for 3D printing and has a tensile strength of 2.1 MPa and an elongation of 134%. Graphene was used as a filler. In this work, the enhanced absorption effect was tested with two different infrared absorbers. As a result, it was discovered that graphene can enhance absorption due to its special structure. With the introduction of graphene, the tensile strength of the composite was 2.8 MPa, and the elongation was 176%. A series of TPEs reinforced with graphene nanoplatelets (GNPs) of varying thickness were produced. SEM analysis showed good dispersion of GNP in the TPE composition. The physical and mechanical properties of the nanocomposites were determined by tensile testing of the samples, which revealed that GNP contributed to a significant enhancement of their hardness and strength properties.^79^

TPEs based on thermoplastic polyester elastomer (TPPE), CB and electrochemically separated graphene were obtained and their frictional properties were studied.^80^ The surface-modified CB/TPPE composite showed approximately fourfold increase in wear resistance and 26% increase in tensile strength compared to the virgin TPPE. Graphene/TPPE composite containing 1 wt% graphene showed 11-fold increase in wear resistance and 43% improvement in tensile strength due to its high dispersion in the polymer matrix. Samples made from nanocomposites have higher friction resistance compared to 3D products based on pure TPPE. Graphene and TPPE nanocomposites have excellent frictional properties. Surface-functionalized and non-functionalized GNPs were melt-blended to prepare TPPE-based composites.^81^ The results showed that the inclusion of GNPs increased the tensile yield strength and tensile strength of TPPE. Since the surface functionalization of GNPs enhances the interfacial interaction, the reinforcing effect is somewhat increased. Also, under the influence of GNP, the elongation at break of the composite increases, which leads to the appearance of a region of highly elastic deformation.

Based on TPE (LDPE and 1,2-polybutadiene in a ratio of 60/40), nanocomposites with functionalized TPE/TiO_2_ titanium dioxide nanoparticles of various natures were obtained and studied.^82^ It was found that the morphology of the composites differs somewhat depending on the specific surface area of TiO_2_. It is shown that TiO_2_ nanoparticles with a large specific surface area and large crystallites lead to the formation of nanocomposites with a porous structure. At the same time, deformation leads to the formation of composites with high strength, resistance to aging, ozone resistance and resistance to water vapor.

In ref. 83, to obtain TPE, PE waste and crushed rubber crumb were mixed in a ratio of 75/25/10 wt%. The influence of γ-rays on the obtained TPE was studied in the range of 100–500 kGy. The mechanical and thermal properties of irradiated composites and their structure were studied using SEM and X-ray diffraction (XRD) methods. The positive influence of γ-rays on the properties of composites has been determined.

To improve the properties of thermoplastic natural rubber (TPNR), graphene oxide (GO) and multi-walled carbon nanotubes (MWCNTs) were incorporated into its composition.^84^ TPNR is composed of a mixture of PP and natural rubber. The mechanical, thermal and electrical properties of TPNR/MWCTs, TPNR/GO and hybrid TPNR/GO/MWCNTs nanocomposites were investigated. The results obtained from the tensile and impact tests showed that the tensile strength and Young's modulus of the TPNR/GO/MWCNTs hybrid nanocomposite were increased compared with those of the TPNR composite and the TPNR/GO nanocomposite, but were lower than those of the TPNR/MWCNTs nanocomposite. The elongation at break decreased with the addition of both nanofillers. The electrical and thermal properties of the samples reinforced with 0.5 wt% MWCNTs were higher than those of the pristine TPNR. The bonds of MWCNTs, GO and MWCNTs-GO were well coordinated, creating a synergistic effect, which was confirmed by SEM images. Due to the presence of this bond, the physical, mechanical, thermal and electrical properties of the nanocomposite were significantly improved.

Chandran *et al.*^85^ prepared TPE by blending nanocomposites based on PP, natural rubber Cloisite 15A (70–30) and TiO_2_. The physical and mechanical properties of the nanocomposites were compared with theoretical approaches such as the Kerner, Guth and Halpin–Tsai models, taking into account the presented fillers. It was found that fillers migrated into the PP phase regardless of their geometric shape, which led to shrinkage of the dispersed phase. The effect of MWCNTs on the physicomechanical and thermal properties of PP/natural rubber blends mixed in two different compositions was studied.^86^ Depending on the ratio of the mixture components, the transition of the composite from a brittle state to an elastic state was determined by the critical concentration of MWCNTs and the composition of the mixture. It was found that the addition of 5 wt% MWCNTs increased the tensile yield strength and impact toughness of the blend. It was shown that the reinforcing effect of MWCNTs was greater in 50PP/50 natural rubber samples than in 80PP/20 natural rubber blends. Dynamic mechanical cyclic analysis confirmed the presence of two glass transition temperatures characteristic of the PP and natural rubber phases.

The effect of GNP on compatibilized dynamically vulcanized multiphase thermoplastic elastomer (TPVs) based on linear low density polyethylene (LLDPE) and reclaimed rubber (RR) was studied based on the obtained experimental data.^87^ The structure of nanocomposites was obtained by the traditional method of mixing components in a melt and studied by various physical methods: transmission electron microscopy (TEM), SEM, DSC, thermogravimetric (TGA) and dynamic mechanical thermal analysis. The physical, mechanical and rheological properties of the samples were also studied. DSC analysis revealed the role of GNP as an effective structure-forming agent in TPVs nanocomposites. For example, the introduction of GNPs into TPE led to an increase in Young's modulus.

To increase the elastic modulus of asphalt concrete pavements, a dynamically cross-linked thermoplastic elastomer was synthesized.^88^ For this purpose, mixtures of LLDPE and styrene butadiene rubber in a ratio of 80/20 were mixed with bitumen and organically modified clay in the melt mode and then vulcanized with sulfur. The process was carried out in an extruder at a temperature of 160 °C with a screw rotation speed of 120 rpm. To enhance the molecular interaction between the polymer phases and clay-silicate layers, maleic anhydride-grafted LLDPE (PE-*g*-MA) with a maleation degree of 1.0% was introduced into the mixture. Based on the results of X-ray phase and transmission electron microscopy analyses, it was established that the clay-silicate layers were divided into smaller ones, followed by the production of highly dispersed nanocomposites.

## 4. Metal–filled polymer nanocomposites

This section presents the results of research over the past 10 years on the influence of metal fillers on a number of properties of PO-based composites. The paper presents the results of a study of the effect of aluminum concentration on the physicomechanical properties of composites based on high density polyethylene and low density polyethylene.^89^ The properties of metal-filled composites, such as ultimate tensile stress, elongation at break, elastic module, melt flow rate, and heat resistance, were studied. According to the data obtained, the loading of aluminum into the composition of low density polyethylene contributes to a monotonic increase in the ultimate tensile stress and the elastic module. When aluminum is loading into the composition of high density polyethylene, on the contrary, a natural decrease in the ultimate tensile stress and elongation at break of the composites is observed. It is shown that when using a compatibilizer, which is polyethylene modified with maleic anhydride, a significant increase in the ultimate tensile stress of high-density polyethylene composites is observed. A schematic representation of the structure of composites with an interpretation of the probable mechanism of hardening of the material in the presence of a compatibilizer is given. It is shown that the crystallinity of the initial polyethylene has a significant effect on the hardening effect of composites. Electron microscopic micrographs of the structure of a filled composite without and with compatibilizer are given. A comparative assessment shows that in the presence of a compatibilizer, aluminum particles are in the bulk of the polymer matrix, *i.e.* are not in an isolated state. It is assumed that HDPE macrochains free of maleic anhydride (MA) are involved in the formation of crystalline formations, and small sections of macrosegments containing polar groups of MA are concentrated mainly in amorphous regions and in defects in crystalline structures in the form of passage chains. The concentration of PEMA macrosegments containing MA in the narrow amorphous space of HDPE favorably affects the increase in the adhesive forces of interaction on the surface of aluminum particles, which affects the preservation of the ultimate tensile stress at a relatively high level over a wide range of aluminum concentrations.

Metal–polymer systems are very promising nanocomposites, since they make it possible to obtain materials with various new properties.^90,91^ When using nano-sized metal particles, the quality of PC obtained on their basis differs significantly from the properties of similar materials with micrometer dimensions. Along with the stabilization of the structure of the polymer matrix, nanocomposites are characterized by good technological, physical–mechanical, thermal–physical, electrical and optical properties. The interaction of the matrix with nanoparticles helps to enhance the structure-forming effect, which provides a unique opportunity to obtain nanocomposites with predetermined properties.^92–94^

Thus, metal–polymer nanocomposites are not only multifunctional materials with a unique set of properties, but also convenient systems for targeted control of these characteristics. The study of such materials usually covers several scientific fields and, therefore, they can find their practical implementation in various fields of electronics.^95^ Due to their size and shape, metal nanoparticles have unique chemical, electronic and optical properties, allowing them to be used as one of the main components in the polymer matrix.^96^

Aluminum is a metal that has high strength and corrosion resistance, is easy to process, has good thermal and electrical conductivity, and can be recycled. Mixing aluminum powder (AP) with polymer improves physical and mechanical properties such as hardness, dimensional stability and thermal conductivity.^97,98^ In works ref. 99 and 100, the influence of the amount of AP on the physical, mechanical (strength, elongation at break, hardness, elasticity) and rheological properties of a composite obtained on the basis of HDPE was studied. The amount of filler was taken in the range of 1–20 wt%. It was found that if the introduction of 1 and 2 wt% AP leads to a slight improvement in the physical and mechanical properties, then the introduction of 5 and 10 wt% AP gives results similar to the properties of the original HDPE. Analysis of composites with a filler content of less than 5 wt% shows that the AP particles are uniformly distributed in the polymer matrix. As a result of the low compatibility of AP with the polymer matrix, a slight decrease in tensile strength and Young's modulus was observed.

**Fig. 4:**
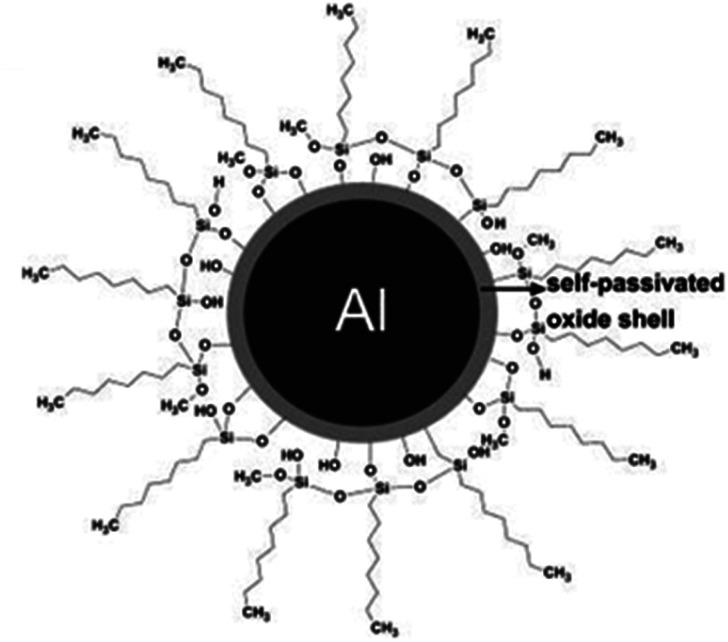
Schematic representation of surface-modified aluminum nanoparticles.^104^

Nor Fasihah Zaaba *et al.*^101^ studied the effect of several metals (Al, Cu, Fe, Ni) on the elongation, thermal and electrical conductivity properties of HDPE-based composites. The authors in their previous studies^102^ stated that AP did not have a negative effect on the properties of HDPE-based composites. In addition, when introducing a metal filler into the HDPE matrix, an increase in Young's modulus was observed, and elongation at break of the filled composite was lower than that of unfilled samples. In the presented work,^103^ the main objective was to study the influence of surface modification of Al nanoparticles on the microstructure and physical properties of nanocomposites. For example, it was found that the use of octyltrimethylsilane as a dressing agent increases compatibility at the interface between the polymer and Al nanoparticles (Fig. 4). Modification of the surface of nanoparticles promotes their uniform dispersion in the polymer volume, which is confirmed by the results of SEM analysis.^104^

The physical and mechanical properties of thermoplastic polyurethane (TPU)/Al_2_O_3_ obtained by extrusion were studied. The study showed that the aluminum oxide surface was pre-coated with a dressing agent, aminopropyltriethoxysilane.^105^ As a result, it was discovered that the treated aluminum oxide has a very positive effect on the process of its uniform distribution in the volume of polyurethane (PU). It should be noted that, compared to the original PU, an increase in the value of Young's modulus was observed in its hybrid composite, despite the fact that the value of the tensile strength in the samples did not change. With increasing aluminum oxide content, elongation at break decreased due to an increase in the brittleness of the composite.^106^ In the era of modern civilization, based on advanced technologies in space technology, CMs with high physical and mechanical properties and cost-effective characteristics were obtained. A mixture of AP and silicon carbide was included in the polymer matrix as a filler. They have attracted more attention due to their use as structural components in engineering fields such as transportation, defense and aviation.^107^

In this regard, in work,^108^ hybrid composites consisting of HDPE, Al and an organosilicon compound were obtained using the extrusion method. The formation of the properties of hybrid composites is the result of the specific features of the influence of components in the polymer matrix. The hybrid composite was found to be the most promising structural material due to its ability to provide better adhesion at the phase boundary in the polymer–filler system. The number of particles in the composites was taken to be 10, 20, 30, 40 and 50 wt%. As a result of increasing the number of particles from 10 wt% to 40 wt%, the strength properties of the hybrid composite increased by 32%. However, the inclusion of particles in an amount of 50 wt% resulted in a decrease in the tensile strength of the composite by 37.8%. This is due to the fact that at high filler contents, agglomeration of particles occurs, which increases the likelihood of defects in the crystalline structure of the polymer matrix. If the tensile strength of a hybrid composite is 17–28 MPa, then for a composite based on Al + HDPE this figure is 15–26 MPa. Such high tensile strength is due to the dressing of filler particles in the Al + HDPE composite.

Quasicrystals are complex metallic alloys with a number of interesting physical properties.^109,110^ The main disadvantage that prevents the wide practical application of quasicrystals is the decrease in their tensile strength when their metal content is more than 40 wt%. These disadvantages are eliminated in quasicrystal-filled PC.^111–113^ Thus, in work,^114^ metal–polymer nanocomposites based on LLDPE and Al_65_/Cu_22_/Fe_13_ quasicrystals were obtained in the process of melt mixing. It has been established that as a result of introducing a small amount of fillers, the elastic modulus and strength properties of nanocomposites increase.

The influence of ZnO nanopowder on the physical, mechanical and thermal properties of a composite obtained from PE and PP was studied.^115^ In this work, two types of ZnO were used: Zinkoxyd active and Zano 20. Zano 20 uncoated zinc oxide. Zano 20 from EverCare acts as a ultra violet filter. It is a natural, non-penetrating, photostable mineral sunscreen. It provides the highest level of transparency. The tensile strength of HDPE-based composites showed that both types of nano ZnO do not have a significant effect on the physical and mechanical properties of PC. When used as a nanofiller in PP, only Zano 20 achieved a slight increase in the strength properties of the composites (Young's modulus 6% and tensile strength 7%). Despite the fact that modification of the ZnO surface with stearic acid contributed to improving its compatibility with the PO matrix, this did not lead to an increase in the physical and mechanical properties of the composite.

The results of a study of the effect of the concentration of finely dispersed copper and crosslinking agents on the thermomechanical properties of composite materials based on high density polyethylene are presented. It is shown that the loading of copper increases the softening temperature of composite materials. At the same time, depending on the test temperature, two physical conditions were recorded: solid and viscous. To improve the compatibility of the mixed components, maleized polyethylene was used as a compatibilizer. Dicumyl peroxide and sulfur were used as crosslinking agents. It was found that at a concentration of dicumyl peroxide of 1.0–2.0 wt%, composites from a highly elastic state pass into an irreversible glassy state and lose their ability to viscous melt flow. During sulfuric vulcanization, composites filled with copper powder are characterized by three physical states: solid, highly elastic, and viscous. The optimal concentrations of reacting components are shown, and the temperature ranges of the corresponding physical states for various composite materials are predetermined. Comparative data of derivatographic analysis and thermomechanical tests are presented.^116^

The effect of the concentration of technical carbon on the peel resistance of nanocomposites based on low-density polyethylene, high-density polyethylene, polypropylene, a compatibilizer, and polyolefins filled with 5.0 wt% aluminum and copper is considered. High density polyethylene modified with maleic anhydride and propylene are used as a compatibilizer. The concentration of technical carbon is varied in the limits of 0–40 wt%. It has been established that polyolefin nanocomposites with 5–15 wt% technical carbon exhibit relatively high peel resistance. The main factors affecting the formation of an adhesion contact have been found.^117^

In work,^118^ the physical, mechanical and morphological properties of elastomer–AP mixtures were studied. The metal filler was introduced into the elastomer composition in an amount of 60–64 wt%. The tensile strength and elongation of the obtained composites were investigated. It has been shown that increasing the amount of AP leads to an increase in tensile strength and a decrease in elongation. The introduction of a metal filler reduces the elasticity of the rubber matrix and, as a consequence, leads to a decrease in the elongation of samples, an increase in hardness and tensile strength.

In a number of studies,^119–122^ the influence of Al and Al/Ni hybrid fillers on the physical, mechanical and electrical properties of epoxy resin was investigated. The obtained results showed that the samples containing 20 wt% Al had the highest compressive strength, but the maximum electrical conductivity was obtained in composites containing 30 wt% AP. When AP was replaced by Ni in different proportions in Al/epoxy resin composites, a slight decrease in conductivity and tensile strength values with a simultaneous increase in hardness was observed in the samples.

The properties of thermoplastic elastomers obtained by mechanochemical synthesis have been developed and studied. Composites of PO (PP and LDPE) filled with elastomers and various metal nanooxides have been obtained.^123,124^ It has been shown that the inclusion of a small amount of fillers in the composition of the composite does not change its degree of crystallinity and dielectric constant, but causes a decrease in the elastic modulus. The size of the nanofiller significantly affects the physical and mechanical properties of the composite. Thus, the inclusion of a metal-containing nanofiller in PP or LDPE/EPDM composites leads to the formation of a fine-spherulitic structure, which has a positive effect on their physical and mechanical properties. The influence of zinc and copper oxide on the properties of nanocomposites based on isotactic PP and maleinized LDPE was studied using X-ray diffraction and thermogravimetric analysis methods. Improvement in the thermal-oxidative stability, strength and rheological properties of the obtained nanocomposites was found.^125,126^ The influence of Cu powder content on the structure and properties of LDPE was studied.^127^ It has been shown that the inclusion of up to 3 wt% fillers increases the thermal stability of HDPE, at least partially. However, no significant difference was observed for LDPE samples, which is probably due to the lower degree of crystallinity of the polymer matrix.

In another study,^128^ the effect of copper powder on the structure and properties of LDPE and LLDPE was investigated. The DSC results showed that the amount of Cu has little effect on the melting temperature of LDPE and LLDPE. According to the results of the analysis of the enthalpy of melting of the samples, the increase in the crystallinity of PE by Cu particles is due to the fact that they exhibit the properties of a nucleating agent in the polymer matrix. Compared to unfilled polymers, composites generally have lower physical and mechanical properties (except for Young's modulus).

## 5. Development and research of electrically conductive polymer composites based on polyolefins

**Fig. 5:**
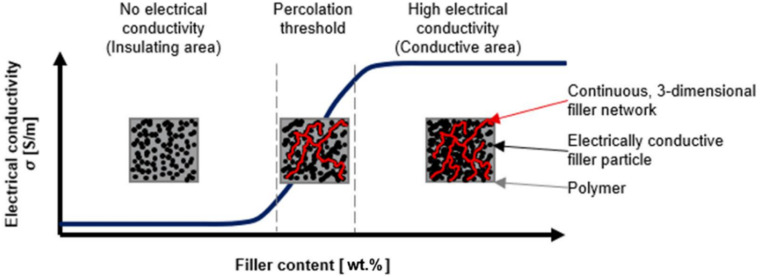
The mechanism of electrical conductivity in polymer nanocomposites depending on the amount of introduced filler.^132^

Most polymeric materials are dielectrics with a resistivity of 10^10^ to 10^15^ Ω m. To increase the electrical conductivity of polymers, highly conductive solid fillers are introduced into their composition – TU, graphite, nanotubes, carbon–graphite fibers or metals. When using carbon fillers, it is possible to obtain a material with a specific resistance of 10^−3^ Ω m, and the use of gold and silver powders allows the specific resistance to be reduced to 10^−6^ Ω m, which is comparable to the conductivity of metals. Electrically conductive polymer composite materials have a number of advantages over metal conductors, the ability to regulate electrical conductivity over a wide range (*ρ* = 10^6^ ÷ 10^−3^ Ω m): the possibility of obtaining products of complex configuration during the processing process, elasticity, corrosion resistance, low density, low cost, *etc.* In addition, they can replace non-ferrous and precious metals such as Cu, Pb, Al, Ag.^129,130^ Many of these polymer composites are characterized by an S-shaped dependence of electrical conductivity on filler content.^131^ Even a slight increase in filler content results in a sharp increase in electrical conductivity. Fig. 5 shows the classic shape of the electrical conductivity curve of a polymer composite depending on the amount of filler. As can be seen from this curve, a sharp change in the number of conductive particles causes coagulation of these particles, which contributes to the formation of conductive chains in the interspherulite space and an increase in the electrical conductivity of the composites. In other words, a qualitative change in properties is observed in the composite, *i.e.* a transition from a dielectric to a conductor. The critical amount of filler at which chain electron conductivity is formed with a subsequent sharp increase in electrical conductivity is called the percolation limit. In the production of electrically conductive nanocomposites, the amount of filler introduced into the polymer matrix is one of the important aspects. Thus, the amount of filler used should be small, but at the same time it should provide sufficient electrical conductivity in the composite. Otherwise, the processing of the mixture becomes difficult and the physical and mechanical properties of the composite deteriorate.

There are several ways to reduce the percolation limit when using conductive fillers in polymer matrices. This is primarily due to the use of additives that allow the optimization of the processing conditions of the composite, taking into account the size of the particles and the uniformity of their dispersion in the polymer volume.^132,133^ As can be seen from Fig. 5, there are three stages of electrical conductivity. At low filler contents, conductive nanoparticles behave as islands unconnected to each other in the polymer matrix.^134^ At this stage, the electrical conductivity of the composite corresponds to the conductivity of the polymer matrix used. As fillers are added further, the particles become more tightly packed and are likely to be in greater contact with each other. At a critical amount of fillers, known as the percolation limit, most particles are in contact with neighboring particles, forming continuous conducting chains. In this case, an electric charge can flow through the polymer without encountering high resistance, thereby providing chain electron conductivity.

Lightweight, stretchable, thermally and electrically conductive nanocomposite materials have attracted considerable scientific interest due to the urgent need for their use to reduce electromagnetic wave pollution (connecting to the 5G era).^135^ In this case, self-healing nanocomposites based on carboxylate nitrile butadiene elastomer (XNBR) were fabricated in the melt mode by sequentially mixing it with MWCNT. Metal–ligand complexes in the XNBR elastomer chain provide feedback that promotes their self-healing. The higher component ratio of MWCNTs helps form interconnected conductive pathways within the XNBR matrix. The nanocomposite with MWCNTs (15 phr) provided excellent EMI shielding efficiency of −27.4 dB while maintaining thermal conductivity at 0.85 W m^−1^ K^−1^. Under harsh practical and environmental test conditions, the nanocomposites showed little change in electromagnetic efficiency, demonstrating their effectiveness in outdoor applications. These ultra-light, self-healing, recyclable MWCNT/XNBR EMI shields are promising materials for next-generation super-elastic and portable smart electronics.

MXene-based elastomer composites that shield electromagnetic radiation are inspiring candidates for ensuring reliable performance of stretchable and wearable electronic gadgets.^136^ However, creating efficient stretchable elastomer/MXene composites with high EMI shielding efficiency, thermal management and processability is challenging. To simultaneously enhance the multifunctional performance of elastomeric composites, 1-(3-aminopropyl)imidazole crosslinked and grafted XNBR/MXene zinc oxide composites were prepared by forming a continuous conductive nacreous network using a simple two-step melt mixing strategy. This enables the composites to exhibit excellent shielding efficiency of −27.4 dB (in the 8.2–12.4 GHz frequency range) and thermal conductivity of 1.24 W m^−1^ K^−1^, while maintaining electrical conductivity of 0.5 S cm^−1^, room temperature self-healing ability of 46.8%, and 100% recyclability with desired tensile strength and elongation. It is noteworthy that the continuous conductive network remains unchanged even after repeated processing, stretching, bending, long-term exposure to sunlight and chemical treatment, which proves its comprehensive chemical and mechanical performance. Such long-term deformations maintain shielding efficiency at more than 95%. Taken together, the developed multifunctional elastomer composites provide the conditions for creating high-performance elastomer composites on their basis with high shielding efficiency and the ability to self-heal, thermally regulate and be recyclable.

Harmful disruptions are a result of the miniaturization and enhancement of contemporary electronics and communication.^137^ Self-healable, flexible, and lightweight elastomeric electromagnetic wave absorbers have replaced metal-based EM wave reflectors as state-of-the-art. Intending to boost the elastomeric nanocomposites' several functions, Herein, we prepared self-healable, flexible, and lightweight ZnO-XNBR/RGO nanocomposites with excellent thermal management and EMI shielding performance. A 1 mm thick nanocomposites film with DC electrical conductivity of 0.02 S cm^−1^, thermal conductivity of 0.75 W m^−1^ K^−1^, a self-healing capability of 54.7%, 100% recyclability, excellent flexibility, and mechanical performance has been recorded with an EMI SE of −34.2 dB in the X-band (8.2–12.4 GHz). Additionally, the uninterrupted conductive network remains unharmed by recycling, expanding, flexing, prolonged exposure to natural light, as well as chemical treatment, justifying its overall mechanical and chemical performance. These extended distortions exhibit greater than 90% retention of their shielding effectiveness. In combination, the properties of our diversified elastomeric nanocomposites, including their superior shielding capability, self-healing capability, thermal management, recycling ability, and outstanding mechanical performance, provide an important guideline for developing robust elastomeric nanocomposites.

As mentioned above, CB and graphite are widely used as readily available and cost-effective fillers that provide electrical conductivity to polymers. Despite a number of results obtained in this area, the general concept of filling non-polar polymers with carbon nanofillers has not yet been fully studied in the literature. The specific electrical resistance of CB is very low, from 10^−2^ to 10^−3^ Ω cm^−1^, which has opened up new promising opportunities for their widespread use as electrically conductive fillers.^138^

The introduction of CB nanoparticles into a polymer matrix is a very effective way of forming PO-based nanocomposites with comparatively high physical, mechanical and thermal properties. The unique properties of CB make it an ideal reinforcing agent in polymer matrices. In addition, the lack of sufficient compatibility of CB with non-polar PO creates difficulties with uniform dispersion of filler particles in the polymer matrix, thereby limiting its practical application for the creation of composites for structural purposes. Thus, carbon nanofillers tend to form clusters, as a result of which the level of adhesive contact of PO macrochains with the specific surface of particles decreases, contributing to the deterioration of the properties of nanocomposites obtained on their basis. To increase the efficiency of using the advantageous structural features and characteristics of nanocarbon, it is necessary to provide conditions for the formation of a full-fledged interaction between the nanoparticle and polymer macrochains in the interphase amorphous space. The use of solution technology does not allow achieving the goals of nanomodification, as a result of which the properties of such a composite become significantly lower than the expected theoretical results. Thus, poor adhesion in the polymer–filler interphase space prevents the creation of a sufficiently strong adhesive contact between the polymer matrix and the nanofiller.^139,140^

Markov *et al.*^141,142^ investigated the effect of the amount of CB grade UM-76 (average particle size ∼20 nm) on the electrical conductivity of a composite based on a mixture of ultra-high molecular weight polyethylene (UHMWPE) (molecular weight ∼7 million). The main objective of the work was to obtain a self-regulating electrically conductive heat-resistant polymer material with an optimal set of thermoelectric properties. It has been shown that the inclusion of UHMWPE in the composite affects the properties of LDPE, strengthening its supramolecular structure. As a result, the negative influence of electrical resistance on the negative temperature coefficient is reduced. In addition, the material can maintain its geometric shape at high temperatures. This may make it possible to eliminate the stage of chemical structure formation when producing self-regulating polymer heating elements. Based on the study of rheological, physical–mechanical, thermal–physical and electrical properties of electrically conductive composites modified with UHMWPE, it was established that an interphase layer resistant to thermal degradation is formed in the LDPE + UHMWPE mixture. It is shown that the obtained polymer nanocomposite containing 30–40 wt% UHMWPE has good operational and self-regulating properties. Based on the obtained results of the study, it was established that composites based on LDPE containing CB and 30 wt% UHMWPE can be processed by extrusion and injection molding, and samples containing 40 wt% UHMWPE can only be processed by pressing. At higher UHMWPE content, composites generally lose their ability to be processed.

**Fig. 6:**
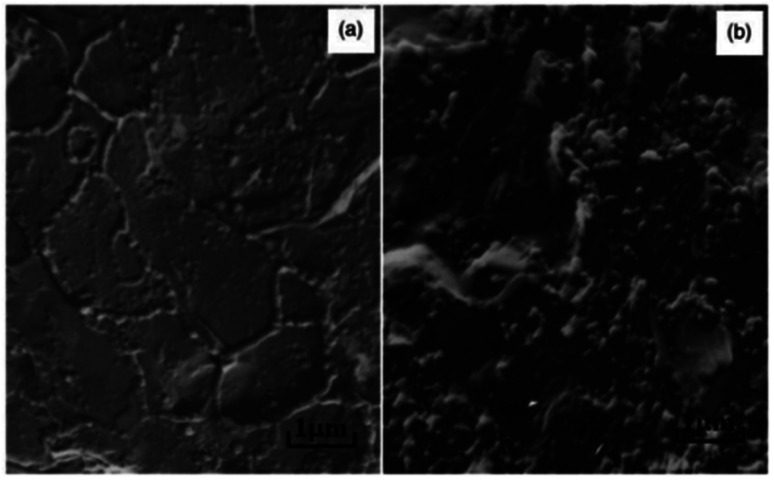
Electron microscopic images of HDPE + 5.0 wt% CB (a) and HDPE + 20 wt% CB (b) nanocomposites. Magnification ×15 000.^143^

In our work^8–12^ we showed that during the cooling and growth of crystalline formations in PO, some of the filler nanoparticles participate in the formation of heterogeneous “nucleation” centers, while the remaining majority of the particles are displaced into the interspherulitic amorphous region. The accumulation of filler particles in the interspherulite region leads to an increase in its density and, as a consequence, to an increase in the probability of the formation of an electrically conductive chain structure. This is confirmed by the results of a unique electron microscopic analysis of composite samples, according to which, at a 5.0 wt% content of CB nanoparticles, chain-like electrically conductive structures are formed in the narrow interspherulitic space of HDPE (Fig. 6a). With an increase in the CB content to 20 wt%, the density of the interspherulitic region increases so much that electrically conductive clusters are formed (Fig. 6b), maintaining the electronic conductivity of the composites at a high level.^143^

**Fig. 7:**
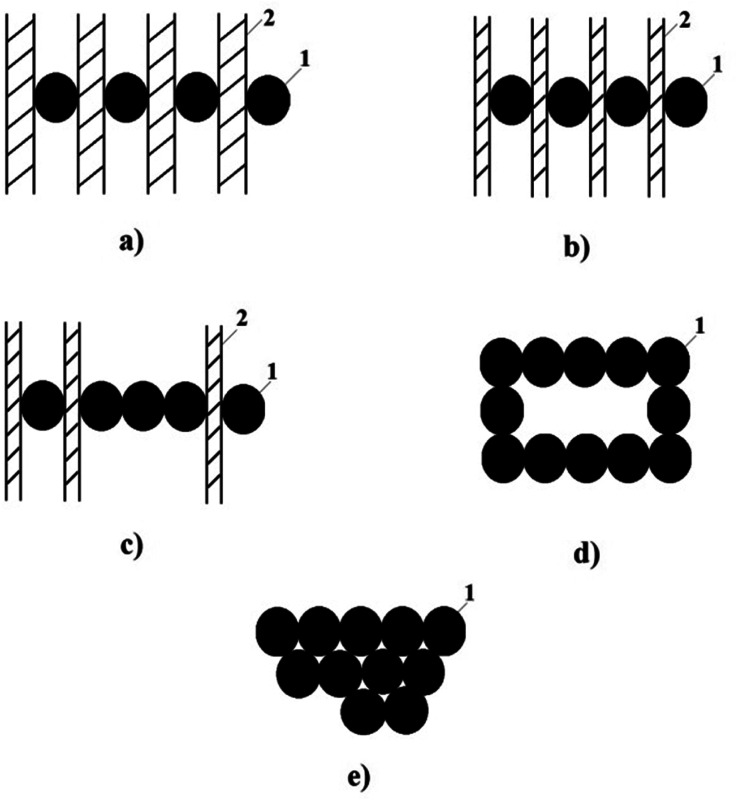
Schematic representation of options for the formation of electronic and tunnel conductivity in nanocomposites based on polyolefin CB: (a) nanoparticles (1) separated by a thick dielectric layer (2); (b) nanoparticles (1) separated by a thin layer of dielectric (2); (c) mixed – electron (1) and tunnel (2) conductivity; (d) chain clusters; (e) nanoparticle agglomerates (1).^143^

For greater clarity, Fig. 7 shows a schematic representation of the proposed variants of the mechanism for the formation of the chain structure of nanoparticles in the interspherulitic space.^143^ It is quite obvious that tunnel conductivity occurs through the dielectric layer, and electronic conductivity, as a rule, becomes possible as a result of direct contact of CB nanoparticles. Therefore, there is reason to believe that, depending on the concentration of CB, one or another conductivity mechanism will predominate. According to the schematic diagram shown in Fig. 7a, the process of formation of an electrically conductive structure in the interspherulite space of a HDPE-based nanocomposite begins even at a CB concentration of 1.0–3.0 wt%. In this case, a chain structure of CB nanoparticles separated by a layer of polyolefin dielectric is formed in the interphase region. This structure provides predominantly tunnel conductivity (Fig. 7a). The fact is that in the process of growth of crystalline formations, not only nanoparticles are displaced into the interspherulitic space, but also segments of the macrochain containing polar groups in the composition of the compatibilizer – maleinized HDPE (PEMA) and maleinized PP (PPMA). As a result, a situation is created where the accumulation of polar groups in the interspherulite space creates favorable conditions not only for improving the compatibility of the mixed components of the mixture, but also for a significant increase in the adhesion of polar macrochains on the surface of nanoparticles. The authors believe that with an increase in the concentration of CB, the thickness of the dielectric layer between the nanoparticles will decrease (Fig. 7b). Finally, a further increase in the CB content will lead to an increase in the probability of direct contact between nanoparticles and the formation of mixed conductivity: electronic and tunnel (Fig. 7c). In this case, the probability of the formation of “chain structures” with a predominance of electronic conductivity increases. In all likelihood, when the CB content in the HDPE composition reaches 10 wt%, the formation of “chain clusters” occurs, in which, solely due to electronic conductivity, the maximum value of the electrical conductivity of nanocomposites is achieved (Fig. 7d). With a further increase in the CB content up to 20 wt%, the electrical conductivity value remains unchanged, provided that the electronic conductivity is maintained. This variant corresponds to the formation of agglomerates of nanoparticles according to the scheme in Fig. 7e.^143^

**Fig. 8:**
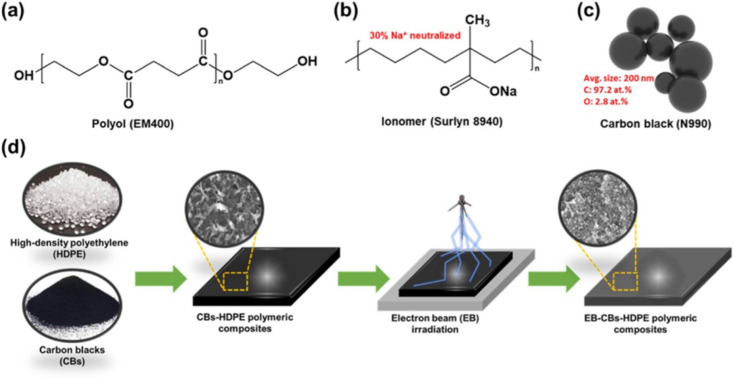
Schematic illustration of the process of manufacturing electron-irradiated CB + HDPE nanocomposites. Chemical structures of (a) polyol (EM400), (b) ionomer (Surlyn 8940), and (c) CB (N990). (d) HDPE.^144^

Analysis of LDPE/CB composites showed that carbon black makes a decisive contribution to improving the physical and mechanical properties at all its contents in the polymer matrix. The introduction of 25 wt% CB leads to an increase in the thermal destruction temperature of the LDPE/CB composite by 76%. The strength properties of LDPE/CB composites are 10% higher than those of the original LDPE. Polymeric materials with positive temperature coefficients (PTC) are considered promising materials for the production of electrical heating elements for use in many areas of electronics. Polymer composites with PTC have several advantages over ceramic or metal oxide composites, including low electrical resistance at room temperature, high flexibility and low cost.^144^ However, the instability of the electrical resistance of composites and the difficulties arising from their secondary processing during the extrusion process create certain limitations for the practical application of composites with PTC. HDPE-based nanocomposites filled with CB in the presence of polar additives – compatibilizers – were subjected to fast electron irradiation with the aim of using them as materials with PTC.^144^ The authors found that electron-irradiated nanocomposites exhibited a PTC, while non-irradiated samples exhibited a negative temperature coefficient (NTC). In addition, the samples containing the ionomer were characterized by a high PTC value of 3.01 Ω cm, which is approximately 35% higher than that of the nanocomposites not containing the ionomer. HDPE/CB nanocomposites are schematically shown in Fig. 8. The obtained results show that the introduction of polar additives and simultaneous irradiation of samples play an important role in improving the PTC. The authors^144^ believe that the obtained results provide important information for the development of carbon–polymer nanocomposites with PTC in various self-regulating heating devices.

In the presented work,^145^ Gan Yan mixed CB with carbon nanotubes (CNTs) and added them to natural rubber to obtain a composite material. The synergistic effect of using a mixture of CB with CNTs, with the formation of a three-dimensional chain structure of electrically conductive circuits in the polymer matrix, was analyzed. The results show that the three-dimensional structure of the “grape bunch” type formed by the synergistic effect of CB and CNT leads to improved vulcanization and physical and mechanical properties of composites, as well as a significant decrease in specific volume resistance. Typically, when mixing 40 parts of CB and 4 parts of CNT with a rubber matrix, the composite has the best set of properties, with the volume electrical resistance being 9.25 × 10^5^.

In work,^146^ the influence of the amount of CB on the strength, electrical and morphological properties of composites based on ER was studied. Epoxy composites were obtained by mixing bamboo stem, hollow oil palm fruit and shell in a certain ratio of CB to ER. By adding 5 wt% filler to the samples, the strength and electrical properties of the composites improved.

Materials that change their electrical resistance during deformation can be used in various areas of electronics, for example, as sensors. For this purpose, the authors^147^ Dionatas Hoffmann Andreghetto *et al.* obtained electrically conductive composites by introducing CB nanoparticles into the epoxy resin. The main goal of the work was to obtain electrically conductive films with piezoresistive properties that could be used as sensors with low deformation. At the same time, an important issue in obtaining films was the determination of a selective solvent. The best solvent for this composite was acetone.

As is known, polymer/nanocarbon nanocomposites have been widely used in the aviation industry recently. In ref. 148, studies were conducted on multifunctional nanocomposites, polymer/nanodiamond, polymer/fullerene and polymer/CB for use in aeronautics. Nanocomposites have been prepared by various methods. The use of nanoreinforcement method for engineering properties of aircraft systems has opened up wide opportunities for studying the properties and applications of nanocomposites. In this regard, the paper discusses in detail the achievements in the field of nanocomposites. A deep understanding of the structure–property relationships in nanocomposites is necessary for the correct selection and practical use of nanomaterials in specialized areas of the aerospace industry.

The influence of paraffin and various amounts of fillers – CB or a mixture of CB + Zn on the morphology, thermal, physical–mechanical and electrical properties of composites based on LDPE was studied.^149^ Various combinations of polymer, paraffin, CB and Zn fillers in the mixture and the study of the effect of radiation-induced structuring of composites led to a change in their degree of crystallinity and immobilization of macrochains, and consequently, physical, mechanical, thermomechanical and electrical properties.

The paper presents the results of a study of the influence of technological parameters of injection molding on the main physicomechanical properties of nanocomposites.^150^ Technological parameters mean the pressure and temperature of the material cylinder, the temperature of the mold, the holding time under pressure, the position of the sprue in the mold in relation to the part being formed. As an object of research, we used multicomponent nanocomposites based on high-density polyethylene, low-density polyethylene, and an ethylene–hexene copolymer with fillers such as technical nanocarbon and aluminum powder. To improve the technological compatibility of the polymer base with fillers, a compatibilizer was used, which is a graft copolymer of random polypropylene with 5.7 wt% maleic anhydride. In connection with the need to impart antistatic properties and high adhesion strength to the metal surface to nanocomposites, the rationale for the choice of these fillers is given. Such physicomechanical properties of nanocomposites as ultimate tensile stress, elongation at break, flexural modulus, volumetric shrinkage, adhesive strength, electrical conductivity is investigated. The results of a study of the effect of temperature and casting pressure on the ultimate tensile stress and elongation at break of nanocomposites based on polyolefins containing technical carbon, aluminum powder, and compatibilizer are presented. It is shown that with an increase in the temperature and pressure of casting, a natural increase in physicomechanical parameters is observed. The data on the influence of the temperature of the mold in the range of 25–70 °C on the properties of composite materials are given. The theoretical substantiation of the processes occurring during the processing of nanocomposites is given. The influence of the sprue location in the mold (along or across) on the properties of the cast specimens is considered. It has been proven that when the product is positioned along the injected melt flow, the strength and elongation of the samples becomes higher than that of the products located across the sprue. A detailed description of the orientation processes taking place in the volume of the cast product is given.

The results of the investigation of the thermomechanical characteristics of maleinized polyolefine-based nanocomposite materials with different carbon black content are presented.^151^ The high density polyethylene, low density polyethylene and polypropylene were used as polyolefins. Highly structured amorphous carbon black of the Printex XE 2B brand with a nanoparticle size of 20 nm, introduced into the composition of the polyolefin in an amount of 1.0–20 wt%, was used as technical carbon. To improve the compatibility of polyolefins with technical carbon, a compatibilizer was used – high-density polyethylene graft copolymer with 5–6 wt% maleic anhydride (PEMA) brand Exxelor PO1040 and polypropylene graft copolymer with 5–6 wt% maleic anhydride (PPMA) brand Exxelor PO1020. The compatibilizer was introduced into the composition of polyolefins in the amount of 2.0 wt%. An electron microscopic, derivatographic and X-ray diffraction analysis of nanocomposites with different technical carbon content was carried out. Thermomechanical studies were carried out on a Kanavets instrument. It was found that with an increase in the content of technical carbon within 1.0, 5.0, 10, 20 wt%, the regularity of change in the thermomechanical curves undergoes significant changes. At a technical carbon concentration of 10 and 20 wt%, an area as a plateau appears on the thermomechanical curves. The most thermally stable plateau appeared for nanocomposites based on maleized LDPE + PEMA and PP + PPMAwith 20 wt% technical carbon content. New scientific approaches are presented for interpreting the discovered regularities, taking into account modern theoretical concepts of the supramolecular crystal structure of nanocomposites and the interfacial amorphous region.

The authors of ref. 152 and 153 investigated the influence of particle size and amount of four different grades of CB on the electrical conductivity of HDPE-based composites. It was shown that composites filled with CB particles of different sizes have different percolation limits. The larger the surface area and the smaller the particle size of the CB, the higher the permeability and the lower the percolation threshold. The authors showed that HDPE/CB composites had significant PTC and NTC in these studies.

The results of studying the structural features of nanocomposites by electron microscopy, X-ray diffraction analysis, derivatography and stepwise dilatometry are presented.^154^ The kinetic regularities of crystallization of nanocomposites based on Exxelor PE 1040-modified high density polyethylene HDPE + PEMA and CB are considered by the method of stepwise dilatometry: the dependence of specific volume on temperature. Dilatometric studies were carried out in the temperature range of 20–210 °C. The concentration of nanoparticles was varied within 1.0, 3.0, 5.0, 10, and 20 wt%. In the process of studying the temperature dependence of the specific volume of nanocomposites, it was found that a first-order phase transition occurs for HDPE + PEMA samples with 1.0–10 wt% CB content at 119 °C, and for a sample with 20 wt% CB at 115 °C. The study of the process kinetics of nanocomposites isothermal crystallization showed that, for nanocomposites with 1.0–10 wt% CB content, the mechanism of the process is characterized by the formation of a three-dimensional spherulite structure with continuously formed homogeneous and heterogeneous nucleation centers. A substantiated theoretical analysis and interpretation of the discovered regularities of the crystallization process and the growth mechanism of crystalline formations is given. Derivatographic studies of nanocomposites were carried out, according to which the features of changes in the thermal–physical properties of nanocomposites depending on the content of carbon black were established. The results of X-ray diffraction analysis of nanocomposites with 20 wt% carbon black content are presented, according to which there is a slight decrease in their degree of crystallinity.

**Fig. 9:**
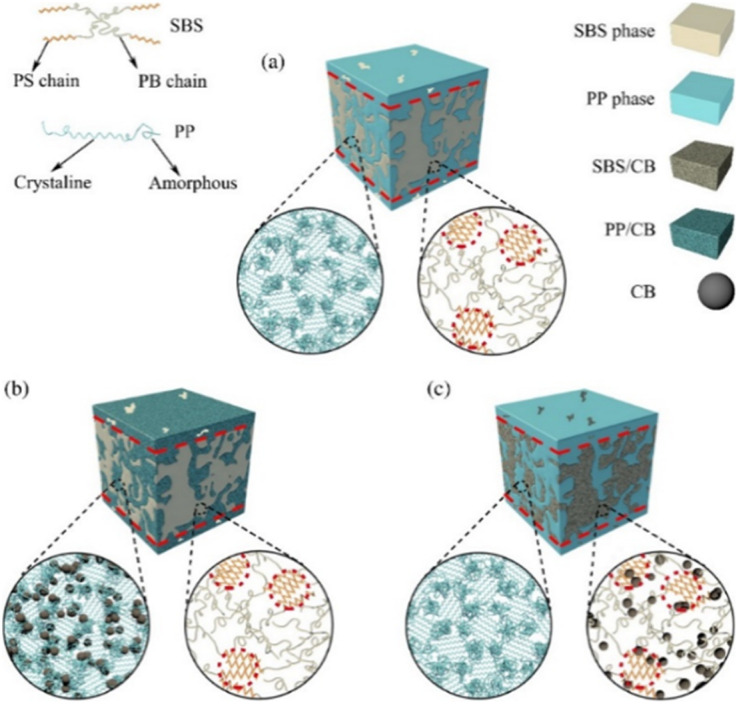
Schematic representation of the distribution of CB in various forms: (a) without CB, (b) CB in PP, (c) CB in SBS.^156^

PP/CB composites were prepared in a Brabender mixer as mixtures with different amounts of CB.^155^ In contrast to work,^156^ 0–5 wt% polyethyleneglycol dimethyl ether was used as a plasticizer in the composite. The plasticizer was used to ensure conditions for uniform dispersion of the CB in the polymer matrix and to reduce the specific surface electrical resistance. However, the plasticizer did not have a positive effect on the specific surface electrical resistance of the composite. On the contrary, the inclusion of a plasticizer in an amount of 5 wt% increased the specific surface electrical resistance by an order of magnitude. At the same time, it was found that the specific surface electrical resistance decreases significantly with an increase in the amount of CB in the polymer matrix in the range of 0–15 wt%. The percolation limit of PP/CB composite is in the range from 5 wt% to 1 wt%. Increasing the amount of fillers leads to a slight decrease in specific electrical resistance. Thermal analysis of the composites showed that the inclusion of CB slightly reduced the crystallization rate of PP. It was found that the concentration of 13.25 wt% CB and 0 wt% plasticizer in the composite is the minimum amount required to sharply reduce its specific surface electrical resistance. PP/styrene–butadiene–styrene (SBS) copolymer was obtained in a 50/50 ratio, with subsequent introduction of CB into its composition by various methods,^156^ which, according to Fig. 9, are numbered as follows: (a) – PP/CB composite mixed with SBS (PC 10S), (b) – SBS/CB mixed with PP (SC10P) and (c) – PP/SBS mixed with CB (PSC10). As a result, the electrical conductivity of the PP/SBS/CB composite increased by seven orders of magnitude, and their specific electrical resistance amounted to 1.57 × 10^1^, 1.68 × 10^2^ and 4.88 × 10^8^ Ω m, respectively. A small amount of CB, participating in the formation of heterogeneous nucleation centers, enters the crystalline phase of PP. The remaining large part of the CB particles during the crystallization process is transferred to the interspherulitic amorphous region, where, according to Fig. 9, the macrochains are located randomly.

An analytical micromechanical model was presented to predict the electrical conductivity and percolation properties of polymer nanocomposites filled with spherical CB nanoparticles.^157^ The proposed model takes into account the electron tunneling effect, the thickness of the interface layer, the size and amount of the filler, as well as the conductivity of the filler, interface layer and polymer matrix. The properties of the interface layer, taking into account the preparation of the polymer matrix and the nanocomposite used and the conductive chain formed as a result of quantum tunneling of electrons and electrical contacts, are the main physical mechanisms that determine the overall electrical conductivity. The presented model accurately describes the measured electrical conductivity of various nanocomposites in a wide range of CB concentrations and takes into account the presence of a sharp transition in the percolation region. It should be noted that the model accurately predicts a wide range of experimentally observed percolation limits for carbon-reinforced polymer composites in the range of 3–30 wt%.

**Fig. 10:**
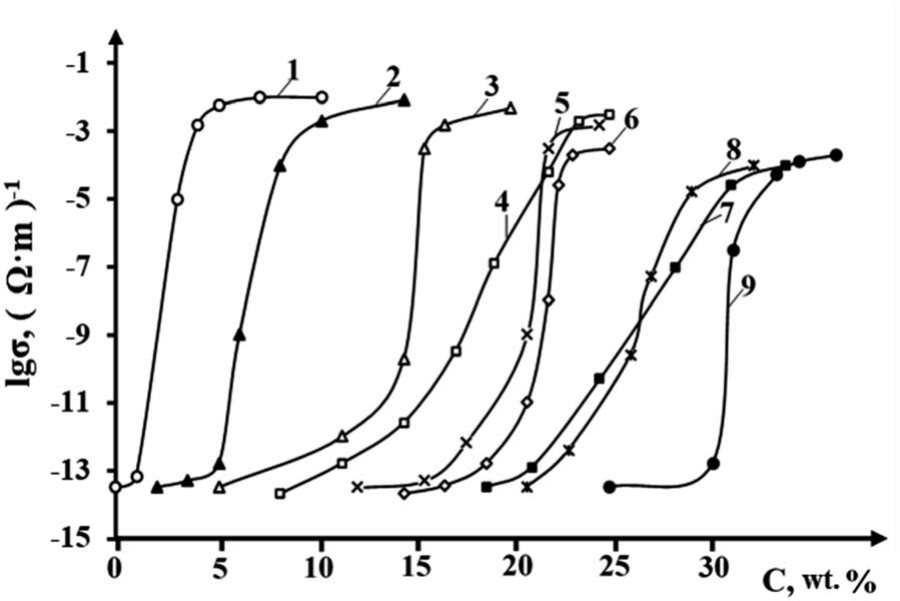
Effect of the content of different grades of CB on the electrical conductivity of HDPE-based composites: (1) – Printex XE 2B; (2) – acetylene soot; (3) – N-550; (4) – P-514; (5) – P-324; (6) – P-803; (7) – K-354; (8) – P-234; (9) – T-900.^158^

The article ref. 158 presents the results of a systemic analysis of the electrical conductivity of nanocomposites based on high-density polyethylene and carbon fillers such as CB and graphite. 13 nano-sized carbon fillers are used. The objective of the study was to select the most effective nanofillers from among the various types used. The effectiveness of nanoparticles was assessed not only by electrical conductivity data, but also by changes in the main physical and mechanical parameters. The properties studied included electrical conductivity, tensile strength, elongation at break and melt flow index. In order to select the most effective electrically conductive fillers, we attempted to evaluate their influence on the electrical conductivity and some physical and mechanical properties of polymer composites using HDPE and a number of CB samples as an example. This polymer is one of the most widely used highly crystalline polymers of the polyolefin class in the world. It should be borne in mind that HDPE, CB and graphite are characterized by insufficiently good compatibility, and therefore a maleated compatibilizer based on HDPE (PEMA) was used as a compatibilizer.^158^ The results of the study are shown in Fig. 10. As can be seen from this figure, the most effective samples are the CB Printex XE 2B (curve 1) and acetylene black (curve 2). The advantage of these samples is that maximum electrical conductivity is achieved at relatively low CB concentrations. For example, if for the first sample the maximum value of electrical conductivity is achieved at 5–7 wt% CB (18–20 nm), then for the second – at 12–14 wt% CB (30–40 nm), the third – 15–17 wt% CB (40–60 nm), the fourth – 23 wt% CB (120–140 nm), the fifth – 21 wt% CB (130–150 nm), the sixth at 22 wt% CB (130–150 nm), the seventh – at 32 wt% CB (160–180 nm), the eighth – at 31 wt% CB (160–170 nm), the ninth – at 33 wt% CB (250–300 nm). The obtained data are of fundamental importance, since they allow us to assert that the difference in the influence of CB on electrical conductivity is due to many reasons. The main ones are dispersion, particle size of the CB and their specific surface area.^159^ In the course of the conducted studies it becomes obvious that all these factors have a positive effect on the formation of electrically conductive chains of nanoparticles. There is reason to believe that during the cooling of the nanocomposite melt, the process of crystallization of HDPE macrochains and the growth of crystalline formations is accompanied by the displacement of nanoparticles into the interspherulitic amorphous region. In this case, the concentration of nanoparticles in the interspherulitic region increases so much that it becomes much higher than its average content throughout the entire volume of the polymer matrix. The concentration of nanoparticles in a narrow interspherulite space becomes one of the main reasons for the formation of chain tunnel or electron conductivity of nanocomposites.

The work^160^ presents the results of studies for obtaining electrically conductive, fire-resistant composites based on HDPE, graphite and some flame retardants. It has been shown that optimum electrical conductivity and flame retardancy, as measured by UL 94, are achieved by modifying graphite-containing HDPE composites with ammonium polyphosphate or aluminum hydroxide. It is shown that the percolation limit of composites based on HDPE and graphite is observed at high content. It can be concluded that an increase in the degree of crystallinity of the binder leads to a decrease in the percolation limit of graphite-filled PE, and, accordingly, with a lower content, to the formation of structurally stable electrically conductive clusters in the composite. Several electrically conductive composites have been obtained, which are characterized as fire-resistant materials. These data allow us to directly predict the values of physical and mechanical properties, electrical conductivity, as well as thermal and, to some extent, fire resistance of composites. Ref. 161 provides information about the spread of graphene in LDPE improves LDPE/graphene nanocompounds' thermal/mechanical/electrical characteristics. Inclusion graphene develops crystallinity; increases the local order of lattice and thermal stability of LDPE/graphene nanocompounds. Percolation occurs with the graphene incorporation of 0.5 wt%. The authors attribute this to the formation of long-range connective carbons is mainly due to the aggregation of graphene throughout the nanoplatelet framework and magnificent structure.

In work,^162^ the electrical conductivity properties of composites based on PP, ER and graphite were studied. PP/epoxy resin/graphite composites containing different amounts of graphite were melt-blended and molded, and then their properties were studied. The obtained results showed that the electrical conductivity increases with the increase in the amount of graphite, and at a graphite content of 70 wt%, the maximum value of the composite bending strength is 54.36 MPa. However, the inclusion of 80 wt% graphite leads to a decrease in this figure to 40.16 MPa. The PP/ER/80 wt% graphite composite has the highest physical and mechanical properties.

## 6. Electrically conductive nanocomposites based on thermoplastic elastomers (TPE)

Conductive TPEs with high elongation and good bending ability have greater potential than traditional rigid conductive PCs in terms of actuators, flexible semiconductors and deformable sensors, and can meet the growing demand for multifunctional conductive materials.^163–168^ TPV is a TPE consisting of a cross-linked rubber phase obtained by dynamic vulcanization.^169^ Such structural features give TPV good technological properties inherent in thermoplastics and the elasticity of conventional vulcanized rubbers. At the same time, TPV, as a rule, has high physical and mechanical properties and is easily processed into products than traditional rubbers and elastomers.^170^ In addition, there is growing interest in conducting various studies in the field of obtaining conductive materials based on TPV, which contribute to the expansion of the areas of their practical use.^171–173^

Ma *et al.*^174^ investigated the filler distribution pattern and electrical properties of dynamically vulcanized PP/EPDM composites and found that the filler particles were mainly distributed in the PP matrix. To achieve a lower percolation threshold and uniform distribution of MWCNTs, a two-step method is required. TPV based on filled CB and bromoisobutylene–isoprene rubber/PP were obtained by two- and one-stage methods.^175^ The mechanical, morphological, electrical, thermal and electromagnetic shielding properties of two-stage and single-stage TPVs were studied. It was found that TPVs obtained by the two-stage method have higher electrical conductivity and better electromagnetic shielding properties. The fact is that the study of morphological properties showed that CB is more evenly distributed in TPV obtained by the two-stage method than in the one-stage method. At a CB content of 16 wt%, both two-stage and single-stage TPVs show good electrical conductivity.

However, two-stage TPV has been shown to have a lower percolation threshold. In ref. 176, a three-stage mixing method was used to prepare linear LLDPE/RR/CNT nanocomposites due to the presence of PE grafted with maleic anhydride as a compatibilizer. It was shown that CNT nanoparticles are mainly distributed in the LLDPE matrix, resulting in the formation of three-dimensional conductive chains, which reduces the percolation threshold.

In another work,^177^ different amounts of MWCNTs were used as filler to obtain new conductive TPEs. Rheological measurements and electrical conductivity tests were carried out to investigate the viscoelastic and electrical percolation behavior of TPE, respectively. It was determined that when unmodified MWCNTs were introduced, the percolation limit of electrical conductivity in these TPEs was 0.34 wt%.^178,179^ The rapid development and increasing use of electronic equipment and devices used in a wide range of industrial, military, consumer and commercial sectors has led to a rapid increase in electronic pollution such as electronic noise and radio frequency interference. Previous studies have shown that metals and their alloys, dielectric ceramics and semiconductors are promising candidates for shielding materials from electromagnetic waves, but their high cost, heavy weight and poor corrosion resistance have limited their application. On the other hand, PCM-based products are easier to manufacture, more cost-effective and more resistant to corrosion. In the presented work,^180^ nanocomposites based on electrically conductive TPEs were obtained and information was provided on the studies conducted to protect them from electromagnetic waves.

Electrically conductive TPEs based on SEBS and graphite/CB were obtained for shielding against electromagnetic waves.^181^ Variations of parameters such as temperature, pressure, rotation speed and mixing time were studied to determine the most suitable processing conditions. The investigated processing parameters did not have a significant effect on the electrical conductivity of SEBS/graphite composites, except for the mixture processed at 230 °C for 15 min. On the other hand, the electrical conductivity of SEBS/CB was found to be dependent on the processing temperature and mixing time. The electrical conductivity, morphology, mechanical, dielectric and rheological properties, and electromagnetic wave shielding efficiency of SEBS/graphite and SEBS/CB composites obtained under the same processing conditions were evaluated and compared. The transition of SEBS/CB insulator to conductor was very sharp and the electrical percolation threshold at room temperature coincided with approximately 5 wt% CB, which was significantly lower than 9 wt% for SEBS/graphite composites. It was found that the amount of CB in the SEBS/CB composite was 15 wt%.

The influence of multilayer graphene (MLG) and few-layer graphene (FLG) on the electrical, thermal and morphological properties of PP/EPDE blends obtained by alloy mixing was studied.^182^ Electrical conductivity studies show that as the amount of graphene increases, the composite transitions from an insulator to a semiconductor. The lower percolation limit is 0.5–1 wt% of MLG for PP/EPDE (60/40). A power-law model was used to describe the electrical conductivity behavior of the obtained graphene-based nanocomposites. The morphology of the composites was studied using SEM, XRD and TEM analyses. It was found that the electrical and thermal properties of the obtained nanocomposites depend on the dispersion of graphene and the size of the dispersed phase. TGA analysis of the nanocomposites shows that the maximum thermal degradation and the onset temperature of thermal degradation coincide with the higher temperatures when graphene is included. At the same time, the inclusion of FLG had a positive effect on the thermal conductivity properties of nanocomposites.

**Fig. 11:**
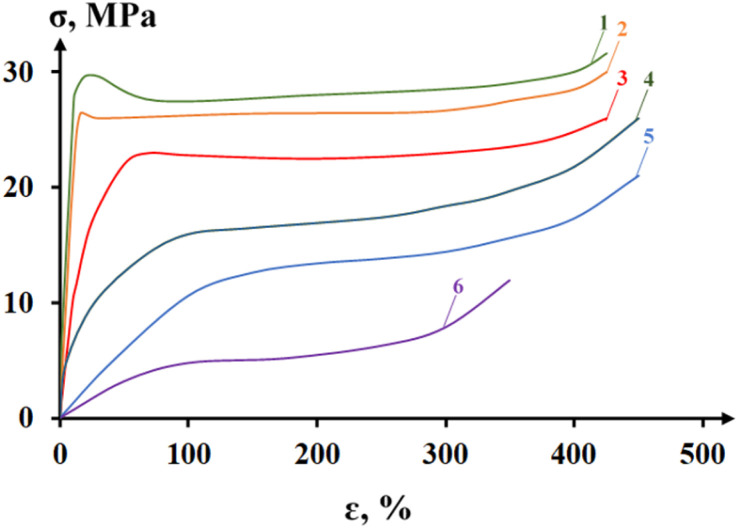
Effect of the content of the elastomer component on the stress–strain curves of polymer blends based on compatibilized HDPE + SCS-30, wt%: (1) – initial HDPE; (2) – 10; (3) – 20; (4) – 30; (5) – 40; (6) – 50.^183^

In work^183^ it was established that with an increase in the content of butadiene–styrene elastomer (SCS-30) in a mixture with HDPE (over 10 wt%), a general tendency towards a decrease in the MFI of polymer mixtures is observed. Apparently, this can be interpreted by the relatively high viscosity of the elastomer, which ultimately affects the reduction of the MFI of the samples. Taking into account that the compatibilizers used have a fairly high melt fluidity, their introduction into the polymer mixture is accompanied by maintaining the MFI at a satisfactory level even at high concentrations of SCS. PEMA and PPMA were used as compatibilizers. The introduction of an elastomer component into the composition of polyolefins eliminates the possibility of destruction of test samples under impact loads. For example, for the original PP, the impact strength was about 10.2 kJ m^−2^. After the introduction of 10 and 20 wt% SCS into the PP composition, the impact strength of the polymer mixtures was 18.4 and 20.3 kJ m^−2^, respectively. It was also established that with an increase in the elastomer content in the composition of polyolefins, not only the strength but also the thermal–physical characteristics decrease. Such a sharp deterioration in properties occurs especially after phase inversion. According to this hypothesis, as the SCS content increases, a moment comes when the thermoplastic polyolefin is transformed from a dispersed medium into a dispersed phase, and the dispersed phase of the elastomer becomes, accordingly, a dispersed medium. This circumstance is due to the fact that in the interphase region the proportion of the amorphous component increases so much that the “long-range order” in the packing of macrochains is disrupted. As a result, in the process of uniaxial stretching of the polymer mixture, the formation of a “neck” becomes impossible, *i.e.* the polymer completely exhibits the properties of rubber. For greater clarity, in Fig. 11, using the example of a HDPE + SCS polymer mixture, the effect of the elastomer component content on the stress–strain curves is examined.

From a comparative analysis of the curves in this figure, it can be established that the introduction of even 10 wt% SCS leads to a decrease in the angle of inclination of the elastic component of strength to the abscissa axis (Fig. 11). As the content of SCS in the HDPE composition continues to increase, the angle of inclination of the curve to the abscissa axis decreases significantly, which indicates a decrease in the value of Young's modulus of the samples. Therefore, starting from this concentration of SCS and higher, samples of the composition begin to exhibit properties characteristic of rubbers, which is expressed in the S-shaped dependence of the stress–strain curves. Using the example of the HDPE + SCS + PEMA polymer mixture, we found that with an increase in the content of the elastomer component, the degree of crystallinity changes in the following sequence: initial HDPE – 82%; 10% SCS – 75%; 20% SCS – 64%; 30% SCS – 52%; 40% SCS – 38%; 50% SCS – 24%. The main physical–mechanical and physical–chemical properties of nanocomposites obtained on the basis of polyolefins, SCS-30 and nanofillers are considered. Vulcanized TPEs with a minimum sulfur content (3.0 wt%) were chosen as the object of study, which contributed to maintaining the melt viscosity at a relatively low level, convenient for their processing. It has been established that, regardless of the type of initial polyolefin, the introduction of nanofillers (CB, Al and calcium stearate) contributes to a significant increase in the strength properties of TPE nanocomposites. The latter circumstance gives grounds to assert that one of the functions of the fillers under consideration in TPE is to enhance their strength properties. Another important indicator is the thermal conductivity of cross-linked TPE nanocomposites, which increases significantly with the introduction of nanoparticles. It is enough to note that if the thermal conductivity of the original polyolefins varies within the range of 0.15–0.22 W m^−1^ K^−1^, then the introduction of the fillers under consideration contributes to a significant increase in the thermal conductivity of TPE nanocomposites to 31–36 W m^−1^ K^−1^, *i.e.*, on average, 200–220 times. In this case, the greatest role in increasing thermal conductivity is given to Al nanoparticles, since its thermal conductivity is 236 W m^−1^ K^−1^, while for dry CB the value of this indicator is only 0.07 W m^−1^ K^−1^.^183^

The work^184^ presents the results of a study of the influence of carbon black nanoparticles of 18–20 nm in size and a compatibilizer on the electrical conductivity of nanocomposites based on polyolefins such as HDPE, LDPE, PP, ethylene–hexene copolymer, and random polypropylene. Maleic anhydride-functionalized high-density polyethylene and polypropylene were used as compatibilizers. The objective of the study was to investigate the mechanism of formation of chain nanocarbon clusters in relation to the objects of study under consideration. It has been shown that the introduction of even small concentrations of carbon black contributes to a significant improvement in the electrical conductivity of polyolefin-based nanocomposites. The concentration of carbon black was varied in the range of 1–30 wt%. It has been established that the introduction of carbon black into the composition of polyolefins makes it possible to obtain electrically conductive nanocomposites of 3 classes: conductors *σ* = 10^−2^–10^−3^ (Ω m)^−1^, semiconductors *σ* = 10^−3^–10^−6^ (Ω m)^−1^, antistatics *σ* = 10^−6^–10^−9^ (Ω m)^−1^. The use of electron microscopy, derivatographic, X-ray structural analysis and thermomechanical studies made it possible to obtain additional information about the mechanism of formation of electrically conductive chain clusters responsible for electron and tunnel conductivity. Using the example of polymer mixtures based on high-density polyethylene and ethylene–propylene–diene rubber, the influence of the degree of crystallinity of the polymer matrix on the electrical conductivity of nanocomposites was studied. The results of the study made it possible to obtain relatively new data for a comprehensive interpretation of the mechanism of formation of chain clusters responsible for the electronic and tunneling electrical conductivity of nanocomposites.

**Fig. 12:**
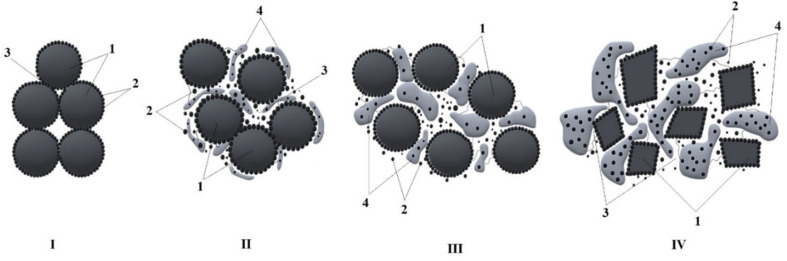
Schematic representation of the redistribution of graphite nanoparticles and the elastomer component (EPDM) in the interfacial region of TPE. (1) – Crystalline region, (2) – nanoparticles, (3) – interphase region, (4) – cross-linked elastomer. Content of elastomer component: (I) – elastomer-free polyolefin nanocomposite; (II) – TPE nanocomposite with 10–20 wt% elastomer content; (III) – TPE nanocomposite with 30–40 wt% elastomer content; (IV) – nanocomposite with 50 wt% elastomer content.^185^

In work,^185^ TPE based on polyolefins and ethylene–propylene–diene rubber and graphite were developed. In a wide concentration range, the regularities of change in electrical conductivity of nanocomposites based on thermoplastic elastomers and nanodispersed graphite were determined. When studying the physical and mechanical properties of TPE nanocomposites, the tensile strength, tensile yield strength, relative elongation, bending strength, heat resistance, melt flow index and Young's modulus were determined. A regularity of changes in the stress–strain relationship for TPE depending on the content of ethylene–propylene–diene rubber has been established. Using methods of derivatography, X-ray phase and SEM analysis, and electron microscopy, studies were conducted to evaluate the structural features of thermoplastic elastomer nanocomposites depending on the ratio of the mixture components used. For a clear interpretation, a schematic representation of the processes occurring in the interphase region of thermoplastic elastomers is provided. Using the X-ray phase analysis method, the regularity of changes in the degree of crystallinity of thermoplastic elastomers depending on the content of rubber and graphite was shown. The fundamental possibility of obtaining flexible electrically conductive materials with predetermined properties by regulating the ratio of components in the composition of thermoplastic elastomers is demonstrated. For greater clarity, Fig. 12 shows a schematic representation of the processes occurring in the interphase region of TPE nanocomposites.

**Fig. 13:**
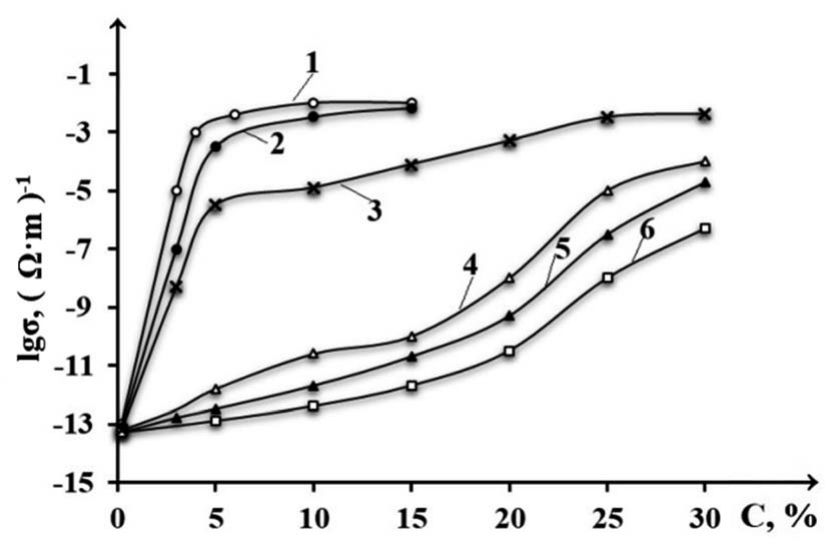
The influence of graphite concentration on the electrical conductivity of compatibilized HDPE + EPDM polymer blends: (1) – HDPE; (2) – HDPE + 10 wt% EPDM; (3) – HDPE + 30 wt% EPDM; (4) – HDPE + 40 wt% EPDM; (5) – HDPE + 50 wt% EPDM; (6) – HDPE + 70 wt% EPDM.^185^

As can be seen from this figure, with an increase in the content of the elastomer component in TPE, an expansion of the interphase region occurs, as a result of which processes are observed that contribute to the disruption of the chain structure of nanoparticles, with a subsequent decrease in the tunneling electrical conductivity of nanocomposites. As can be seen from Fig. 12, the elastomers in the composition of TPE are peculiar “islands” in the dispersed medium of polyolefin. We do not exclude the possibility that during the process of mixing the components of the mixture in the elastomer phase, some of the nanoparticles will become occluded in the composition of the vulcanizates. Another portion of the nanoparticles will likely be distributed in the interspherulitic region to create conductive chains or clusters. Such redistribution of nanoparticles in the composition of TPE will certainly affect the overall reduction of the electrical conductivity of TPE. Therefore, to ensure the required level of electrical conductivity in TPE, it will be necessary to introduce a significantly larger amount of nanofiller. It should also be taken into account that an increase in the elastomer content in the polyolefin composition will contribute to a gradual reduction in the role of “through chains” in the formation of the strength characteristics and plasticity of TPE nanocomposites. In this case, phase inversion will lead to a transition from plastic to highly elastic deformation, characteristic of rubbers.^185^Fig. 13 shows data on the influence of the HDPE/EPDM ratio on the electrical conductivity of nanocomposites at a constant graphite content in polymer mixtures (30 wt%). The concentration of EPDM in the HDPE composition was: 10; 30; 40; 50; 70 wt%. As can be seen from Fig. 13, as the concentration of the elastomer component increases, a noticeable decrease in the electrical conductivity of the nanocomposites is observed.^185^ In a composition containing 10% EPDM in HDPE, the electrical conductivity reaches its maximum {10^−2^ (Ω m)^−1^} at 15 wt% graphite content. With the introduction of 30 wt% EPDM, the electrical conductivity is significantly reduced and reaches its maximum of 10^−3^ (Ω m)^−1^ at 25 wt% graphite content. In a composition with an elastomer concentration of over 30 wt%, phase inversion occurs, when the dispersed phase becomes a dispersed medium and *vice versa*. This is confirmed by curves 4–6, which have a slightly different pattern of change. Moreover, at a concentration of EPDM over 30 wt%, the maximum electrical conductivity at a 30 wt% graphite content becomes lower and fluctuates in the range of 10^−5^–10^−7^ (Ω m)^−1^. This circumstance is due to the fact that as the concentration of EPDM increases from 10 to 70 wt%, a sharp decrease in the degree of crystallinity of nanocomposites in the polymer mixture is observed. Using HDPE as an example, the effect of EPDM on the degree of crystallinity in nanocomposites occurs in the following sequence: (initial HDPE) – 82%, (HDPE + 10 wt% EPDM) – 70%, (HDPE + 30 wt% EPDM) – 54%, (HDPE + 40 wt% EPDM) – 43%, (HDPE + 50 wt% EPDM) – 35% and (HDPE + 70 wt% EPDM) – 18%. It becomes obvious that the lower the degree of crystallinity in the polymer mixture, the lower the probability of forming a chain conductive structure in the amorphous phase.

## 7. Conclusion

Using a wide range of polyolefins (HDPE, LDPE, PP, ethylene–hexene copolymer, ethylene–propylene block copolymer, polypropylene random copolymer, *etc.*), mineral and metal fillers, carbon black and graphite as an example, the real possibility of obtaining electrically conductive materials is demonstrated. The results of the study of the influence of the content and size of filler nanoparticles, the type of polyolefin and the methods of introducing the filler into the polymer matrix on the electrical conductivity and the main physical, mechanical, thermophysical and thermal deformation properties of composite materials are shown.

A list of different types of compatibilizers is provided, which achieve a high degree of dispersion of the mixture components in the polymer matrix. A detailed description of the mechanism of the process of tunneling and electron conductivity in composite materials is given.

The paper examines options for producing thermoplastic elastomers based on polyolefins and various elastomers nitrile butadiene rubber (NBR), styrene butadiene rubber, ethylene–propylene–diene rubber, *etc.* Optimal ratios of polymer components of the mixture have been established, at which highly elastic properties characteristic of rubbers are manifested. Modern theoretical principles and conclusions are used to describe the structure and properties of composite materials.

The possibility of obtaining electrically conductive dynamically vulcanized thermoplastic elastomers with a unique combination of structure and properties is demonstrated. Optimal technological solutions for selecting the temperature regime for extrusion and other parameters for processing dynamically vulcanized composites are presented.

## Abbreviations

AP: Aluminum powder
CB: Carbon black
CM: Composite materials
CNTs: Carbon nanotubes
DSC: Differential scanning calorimetry
EPDM: Ethylene–propylene–diene elastomer
FG: Functionalized graphene
FLG: Few layer graphene
GNPs: Graphene nanoplatelets
GO: Graphene oxide
HDPE: High density polyethylene
LDPE: Low density polyethylene
LLDPE: Linear low density polyethylene
MA: Maleic anhydride
MFI: Melt flow index
MLG: Multi-layer graphene
MWCNTs: Multi-walled carbon nanotubes
NTCs: Negative temperature coefficients
PC: Polymer composites
PCM: Polymer composite materials
PEMA: Polyethylene graft copolymer with maleic anhydride
PO: Polyolefin
POSS: Polyhedral oligomeric silsesquioxane
PP: Polypropylene
PPMA: Polypropylene graft copolymer with maleic anhydride
PTCs: Positive temperature coefficients
PU: Polyurethane
RR: Reclaimed rubber
SBS: Styrene–butadiene–styrene copolymer
SEBS: Styrene–ethylene–butylene–styrene
SEBS-MA: Maleated styrene–ethylene–butylene–styrene copolymer
SEM: Scanning electron microscope
TEM: Transmission electron microscopy
TGA: Thermogravimetric analysis
TPE: Thermoplastic elastomers
TPNR: Thermoplastic natural rubber
TPPE: Thermoplastic polyester elastomer
TPU: Thermoplastic polyurethane
TPVs: Dynamically vulcanized multiphase thermoplastic elastomer
UHMWPE: Ultra-high molecular weight polyethylene
XNBR: Carboxylate nitrile butadiene elastomer
XRD: X-ray phase analysis

## Data availability

All data is obtained from peer-reviewed articles as reported in the references list. No other datasets have been used.

## Conflicts of interest

There are no conflicts to declare.
